# Hyperbilirubinemia as a Marker for Complicated Acute Appendicitis: A Systematic Review

**DOI:** 10.7759/cureus.93197

**Published:** 2025-09-25

**Authors:** Alicja Czyszczon, Bartosz Maczkowski, Aneta Tkaczyk, Marek Drozdz, Wiktoria Boral, Szymon Mikler, Magdalena Mandyna, Aleksandra Przelaskowska, Jan Pulinski

**Affiliations:** 1 Pediatrics, John Paul II Podhale Specialist Hospital, Nowy Targ, POL; 2 General Surgery, Specialist Hospital for Lung Diseases "Odrodzenie" named after Klara Jelska, Zakopane, POL; 3 Traumatology, John Paul II Podhale Specialist Hospital, Nowy Targ, POL; 4 Pulmonology, Florian Ceynowa Specialist Hospital, Wejherowo, POL; 5 Medical Physics, Florian Ceynowa Specialist Hospital, Wejherowo, POL; 6 Surgery, Katowice Oncology Center, Katowice, POL; 7 Pulmonology, Provincial Specialist Hospital in Wrocław, Wrocław, POL; 8 General Practice, Florian Ceynowa Specialist Hospital, Wejherowo, POL

**Keywords:** acute appendicitis, bilirubin concentration, diagnostic biomarker, hyperbilirubinemia, perforated appendicitis

## Abstract

Acute appendicitis (AA) is one of the most common causes of acute abdomen requiring urgent surgical intervention, and early recognition of complicated forms, such as perforation or gangrene, is crucial to reducing mortality and complications. In recent years, bilirubin has gained attention as a potential biomarker of disease severity. The aim of this systematic literature review was to evaluate the diagnostic usefulness of total bilirubin concentration as a marker of complications in AA, with particular emphasis on perforation, and to analyze its effectiveness in differentiating uncomplicated and complicated forms. A systematic search of PubMed, ScienceDirect, and Google Scholar was performed to identify studies exploring the relationship between bilirubin levels and the severity of AA. The literature indicates a correlation between hyperbilirubinemia and perforated appendicitis. Bilirubin has higher diagnostic specificity compared to classical inflammatory markers such as leukocytosis or C-reactive protein (CRP), especially when combined with clinical assessment and imaging studies. Both retrospective and prospective studies suggest that elevated bilirubin significantly increases the risk of complicated disease. Based on the evidence synthesized in this review, hyperbilirubinemia appears to be associated with complicated forms of AA, particularly perforation. While it demonstrates higher specificity than classical inflammatory markers, the available studies emphasize that it should not be used as a stand-alone diagnostic marker. Instead, bilirubin may be considered a supportive parameter, particularly when interpreted together with clinical evaluation and imaging findings.

## Introduction and background

Acute appendicitis (AA) is one of the most common surgical emergencies and remains a leading cause of acute abdominal surgery, particularly in younger patients. It is estimated that 7-9% of the population will experience at least one episode of AA in their lifetime, with a peak incidence between 10 and 30 years of age [1]. Despite significant advances in imaging techniques and surgical treatment, delayed diagnosis is still associated with serious complications, including perforation, abscess formation, sepsis, and increased mortality [2,3,4,5,6]. Approximately 15-30% of AA cases result in perforation, particularly in children and the elderly, who often present with atypical or delayed symptoms [4].

The time from symptom onset to surgical intervention is a key prognostic factor. Longer delays increase the risk of generalized peritonitis, septic complications, and prolonged hospital stay. Therefore, improving the accuracy and timeliness of diagnosis remains a priority in clinical practice [4].

Diagnosis of AA is challenging because its clinical presentation is often nonspecific. Symptoms may overlap with other abdominal conditions, particularly in elderly patients, women, and children. Classic physical findings, such as localized tenderness in the right iliac fossa or peritoneal irritation, are useful but have limited sensitivity [3,5,7,8,9]. Imaging studies play a key role in confirming the diagnosis and identifying complications. Ultrasonography, with a sensitivity of 75-90% and a specificity exceeding 85%, is widely used as a first-line tool, particularly in children and pregnant women [10,11]. Computed tomography (CT) remains the most accurate method, with sensitivity and specificity approaching 97-98%, while magnetic resonance imaging (MRI) offers similar efficacy without radiation exposure, making it suitable for pediatric and obstetric settings [12,13,14,15]. However, these methods may not always be readily available in all healthcare systems, especially in emergency situations.

Laboratory tests, including leukocytosis, neutrophilia, and elevated C-reactive protein (CRP), improve diagnostic yield, but their specificity remains limited [16,17,18,19]. For example, the simultaneous presence of elevated CRP, leukocytosis, and neutrophilia increases sensitivity to nearly 97-100%, but the predictive value of each marker individually is low [20,21,22]. Approximately 19-40% of patients also experience urinalysis abnormalities, such as pyuria, bacteriuria, or hematuria, which may be due to the anatomical proximity of the appendix to the urinary tract, especially when located in the pelvis [23].

Given these limitations, the search for reliable, inexpensive, and easily measurable laboratory biomarkers is ongoing. In recent years, serum bilirubin has emerged as a potential candidate. Several studies suggest that hyperbilirubinemia, particularly elevated direct bilirubin, may reflect endotoxemia-induced liver dysfunction and may serve as a specific marker for complicated appendicitis, including perforation and gangrene [15,24,25,26,27]. Therefore, the aim of this review is to systematically evaluate and synthesize the current evidence on the diagnostic value of serum bilirubin, with a particular focus on its role as a biomarker for complications such as perforation and gangrene in AA.

## Review

Aim of the study

The aim of this review is to synthesize and critically evaluate evidence regarding the diagnostic value of total bilirubin concentration as a biomarker of complications, such as perforation and gangrene, in the course of AA. The study aims to determine whether elevated bilirubin levels can be a useful tool to aid in the differential diagnosis of uncomplicated and complicated appendicitis and to support clinical decision-making regarding urgent surgical intervention.

Materials and methods

This systematic review was conducted in accordance with the PRISMA guidelines.

Search Strategy

A comprehensive search of electronic databases (PubMed, Scopus, Web of Science, and Google Scholar) was performed for studies published between January 2002 and March 2025. The following search terms were applied: (“hyperbilirubinemia” OR “bilirubin”) AND (“appendicitis” OR “appendectomy”). Reference lists of the included articles were also screened to identify additional relevant publications.

Eligibility Criteria

Studies were considered eligible if they (1) were peer-reviewed, (2) investigated patients with AA and reported serum bilirubin levels, and (3) assessed the diagnostic performance of bilirubin in differentiating uncomplicated from complicated appendicitis. Exclusion criteria included studies with small sample sizes, non-peer-reviewed reports, and a lack of full-text availability.

Study Selection and Data Extraction

Two independent reviewers screened titles and abstracts, followed by full-text assessments for eligibility. Any disagreements were resolved by consensus with a third reviewer. From each included article, the following information was extracted: first author, year of publication, study design, sample size, bilirubin cutoff values, sensitivity, specificity, positive predictive value (PPV), negative predictive value (NPV), and area under the receiver operating characteristic curve (AUC), when available.

Quality Assessment

The methodological quality of the included studies was appraised using the Newcastle-Ottawa Scale for cohort studies.

Data Synthesis

Due to considerable clinical and methodological heterogeneity, particularly variability in bilirubin cutoff values, study designs, and outcome measures, a formal meta-analysis was not feasible. Instead, a narrative synthesis was conducted, with results summarized descriptively and presented in both text and tabular form to provide a comparative overview of diagnostic performance.

Results

**Figure 1:**
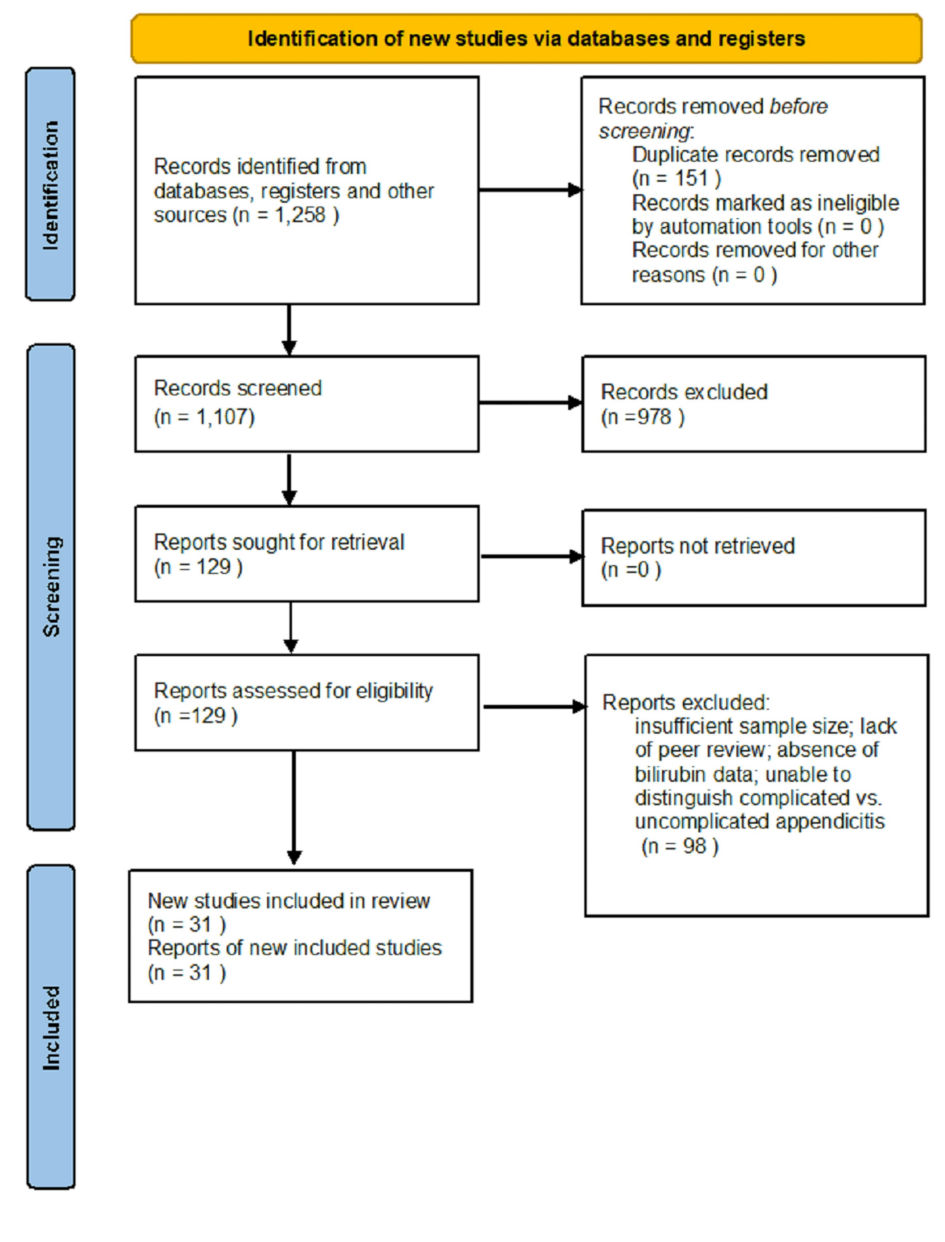
Preferred Reporting Items for Systematic Reviews and Meta-Analyses (PRISMA) 2020 flow diagram illustrating the study selection process.

A total of 1,258 records were identified through database searching and reference list screening. After removal of duplicates, 1,107 records remained. Following title and abstract screening, 978 were excluded as irrelevant. Full texts of 129 articles were assessed for eligibility, of which 98 were excluded for reasons such as insufficient sample size, lack of peer review, absence of bilirubin data, or inability to distinguish between complicated and uncomplicated appendicitis. Finally, 31 studies met the inclusion criteria and were included in the qualitative synthesis (Figure 1).

The methodological quality of the included studies, assessed with the Newcastle-Ottawa Scale (NOS), varied from low-moderate to high (Table 1). Prospective studies (e.g., Ramasamy et al. [28], Anum et al. [29]) generally achieved higher scores (7-8/9), reflecting stronger methodological rigor and reduced risk of bias. By contrast, retrospective studies (e.g., Hong et al. [25], Eren et al. [30]) tended to score lower (4-6/9), largely due to limitations in selection criteria and potential confounding.

**Table 1:** Quality assessment of included studies using the Newcastle-Ottawa Scale (NOS). The NOS evaluates the methodological quality of non-randomized studies in three domains: selection (maximum of four stars), comparability (maximum of two stars), and outcome/exposure (maximum of three stars). The total score ranges from 0 to 9, with higher scores indicating better methodological quality. In this review, studies were classified as high quality (seven to nine stars), moderate quality (five to six stars), or low quality (≤4 stars).

Study	Design	Selection (max 4)	Comparability (max 2)	Outcome/Exposure (max 3)	Total (max 9)	Quality
Sand et al. [27]	Retrospective	★★★	★	★★	6	Moderate
Hong et al. [25]	Retrospective	★★	★	★★	5	Moderate
Ramasamy et al. [28]	Prospective	★★★★	★★	★★	8	High
Anum et al. [29]	Prospective	★★★★	★★	★★	8	High
Hassan et al. [1]	Prospective	★★★	★	★★	6	Moderate
Sreedevi et al. [4]	Prospective	★★★	★	★★	6	Moderate
Eren et al. [30]	Retrospective	★★	★	★	4	Low–moderate

Prospective Studies

Among the prospective studies, several consistently confirmed the diagnostic value of bilirubin as a marker for complicated appendicitis. Ramasamy et al. [28], in a cohort of 378 patients, reported that hyperbilirubinemia >1.3 mg/dL achieved a sensitivity of 80%, specificity of 89%, PPV of 93%, and NPV of 96%, with markedly higher mean bilirubin levels in patients with perforation compared to those with uncomplicated disease. Similarly, Anum et al. [29], analyzing 345 patients, found that bilirubin predicted perforation with very high diagnostic accuracy: sensitivity 90%, specificity 94.9%, PPV 81.8%, and NPV 97.4%. Hassan et al. [1], in 163 patients, demonstrated that bilirubin ≥1.2 mg/dL was significantly associated with gangrenous and perforated appendicitis, with false positives being rare. Finally, Sreedevi et al. [4] observed that while bilirubin >2.0 mg/dL alone had only moderate predictive accuracy (sensitivity 75%, specificity 65%), its combination with CRP substantially improved diagnostic performance, reaching a sensitivity of 85% and a specificity of 75%.

Retrospective Studies

Retrospective analyses provided additional evidence, but with more variability in diagnostic accuracy. Sand et al. [27] showed that bilirubin >1.0 mg/dL was present in 70.1% of patients with perforation, yielding a sensitivity of 70% and a specificity of 86%. Hong et al. [25], in a large retrospective study of 1,195 patients, confirmed that hyperbilirubinemia was an independent predictor of perforation, using a cutoff of 0.85 mg/dL (sensitivity 55.9%, specificity 66.1%, AUC 0.636). Eren et al. [30] demonstrated that diagnostic accuracy improved markedly when bilirubin was assessed in combination with CRP and leukocytosis, achieving values above 90%. Despite these encouraging findings, the retrospective design of these studies introduces a higher risk of bias compared to prospective analyses.

Pediatric and Mixed Populations

A smaller number of studies focused on pediatric or mixed-age populations. Harris et al. [2] reported that indirect bilirubin was particularly sensitive in predicting complicated appendicitis in children, with 100% sensitivity but only 33% specificity, resulting in frequent false positives. Other pediatric studies also highlighted differences compared with adults, suggesting that age-related physiological factors such as hepatic metabolism may influence bilirubin levels [22]. These findings indicate that diagnostic thresholds may need to be tailored for pediatric patients rather than applying uniform criteria across all populations.

Overall Diagnostic Performance

Across all 31 included studies, reported bilirubin cutoff values for predicting complicated appendicitis ranged from 0.85 to 2.0 mg/dL, with most studies adopting thresholds between 1.0 and 1.3 mg/dL [1,4,25,27,28,29]. Within this range, specificity generally exceeded sensitivity, underscoring bilirubin’s value in confirming rather than excluding perforation. Reported sensitivity values varied from 55.9% to 90%, while specificity ranged from 65% to 94.9%. Importantly, prospective studies consistently reported very high negative predictive values (>95%), highlighting that normal bilirubin levels strongly reduce the likelihood of complicated disease.

Taken together, the results demonstrate that hyperbilirubinemia is a reproducible marker of complicated appendicitis across diverse populations and study designs, with prospective studies providing the strongest evidence of its diagnostic utility (Table 2).

**Table 2:** Comparative table of the results of studies on hyperbilirubinemia in acute appendicitis PPV: positive predictive value; NPV: negative predictive value; AUC: area under the receiver operating characteristic curve

Authors / year	Patients (n)	Sensitivity (%)	Specificity (%)	Bilirubin cutoff (mg/dL)	AUC	PPV / NPV (%)
Sand et al. [27] (2009)	538	70	86	>1.0	–	–
Hong et al. [25] (2012)	1195	55.92	66.11	>0.85	0.636	–
Ramasamy et al. [28] (2018)	378	80	89	>1.3	–	PPV 93 / NPV 96
Anum et al. [29] (2022)	345	90	94.91	>1.0	–	PPV 81.82 / NPV 97.39
Hassan et al. [1] (2024)	163	78.17	85.71	≥1.2	–	–
Sreedevi et al. [4] (2025)	200	75	65	>2.0	–	PPV 55 / NPV 80

Discussion

This review indicates that hyperbilirubinemia is a clinically relevant marker of complicated appendicitis, particularly perforation and gangrene. Across multiple studies, serum bilirubin levels were significantly higher in patients with complicated appendicitis compared with those with uncomplicated disease [1,4,25,27,28,29]. Although the reported cutoff values varied between 0.85 and 2.0 mg/dL, most studies consistently demonstrated that the specificity of bilirubin is superior to its sensitivity. This suggests that bilirubin is most useful for confirming the likelihood of perforation rather than excluding it, and its measurement may therefore play an important role in clinical decision-making when combined with other diagnostic tools.

Pathophysiological Mechanisms

The association between hyperbilirubinemia and complicated appendicitis can be explained by well-established biological mechanisms. Perforation of the appendix facilitates bacterial translocation and systemic endotoxemia, most commonly due to Escherichia coli and other enteric organisms. Endotoxins impair hepatocellular transport of conjugated bilirubin into bile canaliculi, leading to intrahepatic cholestasis and elevated serum bilirubin levels, particularly in the direct fraction [24,25]. This mechanism explains why bilirubin demonstrates relatively high specificity compared with conventional inflammatory markers such as leukocytosis or C-reactive protein, which can be elevated in a wide variety of inflammatory conditions. Furthermore, several studies have observed a disproportionate increase in the conjugated fraction, supporting the hypothesis of endotoxemia-induced hepatic dysfunction as a marker of disease severity [28].

Strength of Evidence and Quality Assessment

When critically evaluating the strength of the evidence, it is important to consider the methodological quality of the included studies. Quality appraisal using the Newcastle-Ottawa Scale revealed considerable variability. A minority of studies were prospective, multicenter investigations with standardized bilirubin assessment [28,29], providing robust data with low risk of bias. However, the majority of included studies were retrospective and single-center [25,27,30], which, although often involving large sample sizes, are inherently prone to selection bias, incomplete data, and lack of control for confounding factors such as pre-existing liver disease, hemolysis, or sepsis. These factors may independently influence bilirubin levels and thereby reduce internal validity. Consequently, while the overall body of evidence supports an association between hyperbilirubinemia and complicated appendicitis, the predominance of retrospective studies lowers the certainty of these conclusions.

The findings of this review should be interpreted in the context of the methodological quality of the included studies. While several prospective studies scored highly on the Newcastle-Ottawa Scale, supporting the robustness of their conclusions [28,29], the majority of evidence was derived from retrospective, single-center studies with moderate or low scores [25,27,30]. These studies are more vulnerable to selection bias, incomplete reporting, and lack of adjustment for confounders such as liver disease or sepsis, which may independently influence bilirubin levels. As a result, the strength of the overall conclusions is moderated by the predominance of moderate-quality evidence. Future research should therefore prioritize high-quality, multicenter, prospective studies to validate these findings and reduce the risk of bias.

Heterogeneity in Cutoff Values

Another limitation is the heterogeneity of cutoff values used to define hyperbilirubinemia. Most studies applied thresholds between 1.0 and 1.3 mg/dL, which produced the most consistent diagnostic accuracy [27,28,29]. Others adopted lower cutoffs (e.g., 0.85 mg/dL [25]) to maximize sensitivity, while some used unusually high thresholds (>2.0 mg/dL [4]), prioritizing specificity. These variations strongly affected diagnostic outcomes: lower thresholds increased false positives, whereas higher thresholds increased false negatives. The lack of standardized cutoffs complicates direct comparisons between studies and limits clinical applicability. Standardization of bilirubin thresholds is therefore necessary to enhance reproducibility and to allow integration of this biomarker into clinical practice.

Influence of Study Design and Populations

Study design and patient populations also contributed to heterogeneity. Prospective studies, with predefined bilirubin testing and histopathological confirmation, generally reported higher diagnostic accuracy than retrospective studies [28,29]. Adults formed the majority of the study populations, and in these groups, bilirubin consistently demonstrated good specificity for complicated appendicitis [27,28]. In pediatric and mixed cohorts, bilirubin showed higher sensitivity but lower specificity [2,4,22], reflecting possible differences in hepatic metabolism and immune response across age groups. These findings suggest that a “one-size-fits-all” approach may not be appropriate and that population-specific thresholds or combined diagnostic algorithms may be required.

Overall Interpretation

Taken together, the evidence indicates that hyperbilirubinemia has value as an adjunctive biomarker in the assessment of AA. Elevated bilirubin levels are strongly associated with perforated and gangrenous forms, while normal values have a consistently high negative predictive value, aiding in the exclusion of severe disease. However, the strength of this evidence is tempered by methodological limitations, variability in cutoff definitions, and differences between populations.

Bilirubin testing nevertheless offers several advantages: it is inexpensive, routinely available, and rapidly performed. When interpreted alongside clinical evaluation, standard laboratory markers, and imaging, it can improve diagnostic accuracy and guide urgent surgical intervention [31]. Its role, however, should remain supportive, as no single biomarker can replace the combination of clinical judgment and imaging modalities.

Future Directions

Future research should focus on large-scale, prospective, multicenter trials with standardized bilirubin cutoffs, stratification by age and comorbidities, and integration into composite diagnostic models. Such studies are essential to confirm the clinical utility of bilirubin and to define its role in evidence-based diagnostic pathways for AA.

## Conclusions

AA remains a diagnostic challenge, especially in complicated cases. This review confirms that hyperbilirubinemia is a highly specific biomarker for complicated appendicitis, particularly perforation, and can significantly enhance diagnostic accuracy when combined with clinical assessment and conventional laboratory markers. Although it should not be considered a standalone marker, its measurement can complement standard diagnostics and support physicians in clinical decision-making. Further prospective, multicenter studies are needed to determine the optimal cutoff point for bilirubin concentration and verify its predictive value in various age and clinical groups.

Looking ahead, large-scale, prospective, multicenter studies are urgently needed to determine a definitive cutoff value for bilirubin concentration, to validate its predictive power across different age and clinical groups, and to assess how it can be integrated into broader diagnostic scoring systems together with other laboratory and imaging tests. Such studies will be crucial for confirming the clinical utility of bilirubin and for developing more accurate, evidence-based diagnostic pathways for AA.
